# Artificial Light at Night (ALAN) Is the Main Driver of Nocturnal Feral Pigeon (*Columba livia f. domestica*) Foraging in Urban Areas

**DOI:** 10.3390/ani10040554

**Published:** 2020-03-26

**Authors:** Lucas M. Leveau

**Affiliations:** Departamento de Ecología, Genética y Evolución, Facultad de Ciencias Exactas y Naturales, Universidad de Buenos Aires–IEGEBA (CONICET–UBA), Ciudad Universitaria, Pab 2, Piso 4, Buenos Aires 1426, Argentina; lucasleveau@yahoo.com.ar

**Keywords:** artificial light at night, circadian rhythm, *Columba livia*, Latin America, noise, temporal homogenization

## Abstract

**Simple Summary:**

Artificial light at night is one of the most extreme alterations in urban areas, which drives nocturnal activity in diurnal species. However, the role of artificial light in the nocturnal activity of the Feral Pigeon (*Columba livia f. domestica*) is unknown. The objective of this study is to assess the environmental factors associated with the nocturnal activity of the Feral Pigeon in Argentinian cities. Nocturnal foraging by the Feral Pigeon was recorded in three of four surveyed cities. Artificial light at night was positively related to nocturnal foraging activity in Salta and Buenos Aires. The results obtained suggest that urbanization would promote nocturnal activity in Feral Pigeons. Moreover, nocturnal activity was mainly driven by artificial light, which probably alters the circadian rhythm of pigeons.

**Abstract:**

Artificial light at night (ALAN) is one of the most extreme environmental alterations in urban areas, which drives nocturnal activity in diurnal species. Feral Pigeon (*Columba livia f. domestica*), a common species in urban centers worldwide, has been observed foraging at night in urban areas. However, the role of ALAN in the nocturnal activity of this species is unknown. Moreover, studies addressing the relationship between ALAN and nocturnal activity of diurnal birds are scarce in the Southern Hemisphere. The objective of this study is to assess the environmental factors associated with nocturnal activity of the Feral Pigeon in Argentinian cities. Environmental conditions were compared between sites where pigeons were seen foraging and randomly selected sites where pigeons were not recorded foraging. Nocturnal foraging by the Feral Pigeon was recorded in three of four surveyed cities. ALAN was positively related to nocturnal foraging activity in Salta and Buenos Aires. The results obtained suggest that urbanization would promote nocturnal activity in Feral Pigeons. Moreover, nocturnal activity was mainly driven by ALAN, which probably alters the circadian rhythm of pigeons.

## 1. Introduction

Artificial light at night (ALAN) is one of the most extreme environmental alterations in urban areas, and has been postulated as the main driver of bird foraging and singing at night in diurnal species [1]. Moreover, ALAN affects the orientation of species, attraction to and repulsion from light, reproduction, and visual communication [2]. Several studies focusing on birds, which are the most widely studied animal taxa in urban areas [3,4], have shown increased activity of diurnal species at night in the presence of artificial light [5,6,7,8,9]. Diurnal birds have been recorded singing, foraging, or doing both activities at night [6,8,9,10]. ALAN alters the secretion of melatonin, a hormone related to the biological rhythms in animals [11,12]. The breeding cycle of birds singing at night may be altered, with possible consequences on bird population dynamics [5]. On the other hand, bird foraging at night may have more profound consequences on interspecific relationships. Nocturnal foraging can help to avoid interference competition with other diurnal species or increase the depletion of resources used by nocturnal species [2,6]. Moreover, nocturnal foraging may provide energy reserves and avoid exposure to diurnal predators [13]. Noise has also been associated with nocturnal activity in diurnal birds [14,15].

Studies addressing nocturnal activity of diurnal birds induced by artificial light have increased in the last decade [1,5,10,16]. However, most of them have been conducted in the Northern Hemisphere, with the Blackbird (*Turdus merula*) being the most frequently studied species [8,16,17,18,19]. Although the Feral Pigeon (*Columba livia f. domestica*), which is a cosmopolitan urban dweller [20,21], has been recorded foraging at night in urban areas of Europe [22], the relationship between nocturnal foraging and artificial light has still not been analyzed.

Given the lack of studies about the nocturnal activity of Feral Pigeon, the objectives of this study were 1) to determine the presence of nocturnal foraging by Feral Pigeon in the most highly urbanized areas of four large Argentine cities and 2) to analyze the relationship between nocturnal foraging and artificial light, pedestrian traffic, and car traffic in Buenos Aires and Salta cities. Pedestrian traffic can be considered an indicator of food resources (e.g., human leftovers [23,24]), whereas car traffic is an indicator of noise [25].

## 2. Material and Methods

The presence of nocturnal foraging was evaluated in four of the most populated cities in Argentina (Table 1): Salta, Buenos Aires, Mar del Plata, and Rosario. Surveys were carried out between July and September 2016, corresponding to the austral winter. In Rosario, Mar del Plata, and Salta, surveys were conducted in the downtown area during one single night. In Buenos Aires, due to its bigger size than the other cities, surveys were carried out during three nights, with the pedestrian street being surveyed during one night and two avenues during the other two nights. In Argentina, the downtown area is composed of an urban square surrounded by headquarters, schools, and a church. Surveys were carried out once on pedestrian streets and main avenues; they were walked in search of foraging pigeons, which were recorded by sight or sound. The length of streets and avenues surveyed on foot in each city was proportional to its population size (Table 1). In Buenos Aires, the biggest city, 10 km were surveyed, whereas in Salta, the smallest surveyed city, 1.8 km were surveyed. Due to logistical constraints, surveys started between 33 and 80 min after local sunset and lasted between 36 and 120 min.

In Buenos Aires and Salta, which have at least two records of nocturnal pigeon foraging, an equal number of nocturnal foraging sites and random sites where pigeons were not seen foraging at night were selected; each foraging site had 30 m radius. For example, in Salta, nocturnal foraging pigeons were observed in four different sites, and four random sites with no foraging pigeons were also located. Random sites were located in the most highly urbanized area of each city, and included the pedestrian street and avenues surveyed to record nocturnal pigeons. The area was divided in quadrats of 100 × 100 m, which were numbered. Then, a random number generator was used to select the quadrats without nocturnal foraging pigeons. To ensure independence, sites were separated at least 100 m of one another. In both site types, light intensity, and car and pedestrian traffic, were measured once (Table 2). Car and pedestrian traffic was obtained by counting the number of people and cars passing during three minutes within a 30 m radius. In the pedestrian streets there was no car traffic. Pedestrian traffic was considered a possible indicator of food for pigeons, because of the greater the number of passing people and the greater the chance of pigeons to obtain food from them intentionally or unintentionally. Car traffic was used as an indicator of noise, given that some studies have suggested that noise is a driver of diurnal bird activity at night [14,15]. Light intensity was measured with the Lux Meter app (My Mobile Tools Dev. Lux Meter, Light Meter), using a Sony Xperia M smartphone (Sony Mobile Communications, Tokio, Japan). The smartphone was moved in all directions, 360 degrees, describing an imaginary sphere during five seconds at 1 m height to obtain the mean value of lux, with an error of one lux. Given that the measurements obtained with Lux Meter app were not compared with values obtained with real lux meter indicator equipment, the lux values can be biased and therefore must be used as a guiding reference of luminance levels [26]. 

## 3. Statistical Analysis

The relationship between pigeon nocturnal foraging and environmental variables in Buenos Aires and Salta was analyzed using a generalized linear model (GLM) in R [27]. The response variable was the presence or absence of nocturnal foraging, and a binomial error structure was used. The independent variables were the light intensity (lux), the number of passing cars, pedestrians, and the city. Model selection was performed by running the model with all the environmental variables and gradually removing the non-significant variables until the final model was obtained (*p* > 0.05); significance was tested with the anova function. The final model was compared with the null model through a Likelihood Ratio Test (LRT), and the pseudo-rsquare of the final model was obtained with the function rsquared of the piecewise SEM package [28]. The final model was plotted with the visreg package [29]. There was no significant correlation between the continuous environmental variables (r < 0.70). 

## 4. Results

Nocturnal foraging by pigeons was observed in three of the four surveyed cities (Table 1). Buenos Aires and Salta had the highest number of nocturnal foraging sites (eight and four, respectively), whereas Mar del Plata only had one site, where one nocturnal foraging pigeon was recorded in the pedestrian street. In Buenos Aires, between 1 and 16 pigeons were present in each foraging site (mean = 7.5, standard deviation = 6.07, N = 8), whereas in Salta between one and three pigeons were seen (mean = 1.75, standard deviation= 0.96, N = 4). In Salta, nocturnal foraging was observed only in pedestrian streets, whereas in Buenos Aires it was recorded in the pedestrian streets and the avenues.

**Figure 1:**
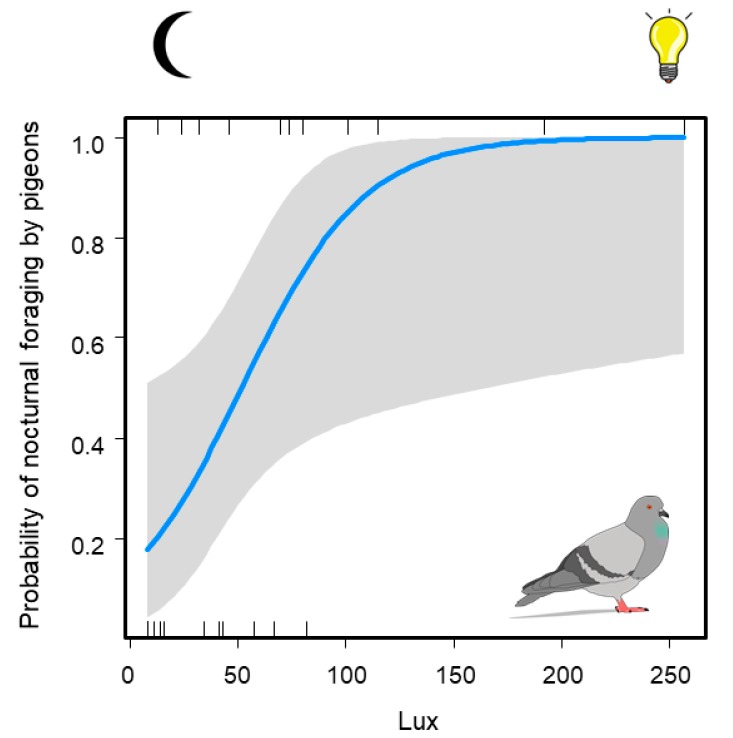
Probability of nocturnal foraging by the Feral Pigeon (*Columba livia f. domestica*) in Salta and Buenos Aires in relation to light intensity (mean lux). The vertical lines indicate the mean lux values of presence sites (above) and absence sites (below). The blue line represents the fitted curve the grey area represents the confidence intervals at 95%.

The final model only included lux as a significant variable related to nocturnal foraging (Table 3; LRT = 8.27, P = 0.004, pseudo-$R^{2}$ = 0.39). There was a significant increase in the probability of nocturnal foraging with increasing light intensity (Figure 1). Light intensity above 100 lux had the highest probability of nocturnal foraging occurrence.

## 5. Discussion

Feral Pigeon was found to forage at night in three of the four surveyed cities, with this activity being mainly influenced by ALAN. The records obtained in the three Argentine cities show that the Feral Pigeon have nocturnal foraging activity in South America and Europe [22]. Other species, most of them Passeriforms and waders, have also been recorded foraging at night in sites with artificial light [5,6,7,8,9,10,16,30,31,32]. However, most of the records belong to the Northern Hemisphere [1].

The present study focused on cities of more than 500,000 inhabitants; the effect of urbanization on nocturnal pigeon behavior needs to be studied in smaller cities. On the other hand, further studies are needed to elucidate why nocturnal foraging of the Feral Pigeon is more likely to occur in some cities and not in others. Possible explanations may be related to the levels of ALAN and the availability of discarded food by humans. Indeed, in three cases of nocturnal foraging in Buenos Aires two people were actively feeding pigeons, and in another case pigeons were foraging on discarded food near a garbage container. Moreover, Feral Pigeon abundance varies with town area and land-use type surrounding the town [33]. Therefore, in those cities with high pigeon density, intraspecific competition may be avoided by nocturnal foraging [1].

Artificial light was the main predictor of nocturnal foraging by pigeons. This result agrees with other studies focused on Passeriforms in the Northern Hemisphere [6,7,8,18,34]. However, our study showed that pigeons extended their activity after sunset, in agreement with previous findings [18,35], whereas other studies only found nocturnal activity just before dawn [7,8]. Unfortunately, the present survey did not cover the entire night; therefore, whether pigeons extended their activity until just before dawn was not determined. Other studies that included 10 species did not find a significant effect of light intensity on bird activity before dawn [16,19]. 

Disagreements among studies may be related to differences in light intensity. For instance, the present analysis spanned a mean light intensity between 8 and 257 lux and was conducted in a highly urbanized area composed of tall buildings and commercial areas. However, Ockendom et al. [16] and Clewley et al. [19] conducted their studies in a less urbanized area, composed of houses with gardens and probably with lower light intensity. On the other hand, studies also differed in terms of the methodologies used, which may have influenced the results. For example, Ockendom et al. [16] used data obtained by citizen science projects, whereas Dominoni et al. [35] used data obtained through light loggers. Moreover, light color also influences bird activity [36]. For instance, Ouyang et al. [37] found that night activity of Great Tits (*Parus major*) increased with white light.

Experimental studies have shown that the increased activity induced by artificial light in birds is related to the reduced expression of melatonin [11]. Melatonin is secreted by the pineal gland and has a central role in the vertebrate circadian rhythm [11,38]. Russ et al. [18] found a significant extension of night activity of Blackbirds under urban conditions with a mean light intensity of 0.44 lux. The results obtained in the present study showed that a mean light intensity above 100 lux had the highest probability of driving nocturnal foraging behavior. 

In this analysis, pedestrian traffic was used as an indicator of food availability for pigeons; however, this variable did not affect their nocturnal activity. Further studies should include other variables indicating food availability, such as number of garbage containers, number of people feeding birds, and presence of nearby restaurants or schools [39]. Moreover, nocturnal foraging by pigeons under artificial light can increase in cities located near the Poles during winter, given the scarcity of natural light. On the other hand, noise has been claimed by several studies as a driver of nocturnal singing in birds [14,15]. However, car traffic did not show a significant relationship with nocturnal foraging in the present study. 

Feral pigeons may have nocturnal foraging behavior to avoid humans, since heavy pedestrian traffic during the day may impair pigeon foraging [40,41,42]. Therefore, pigeons may exhibit nocturnal foraging behavior to minimize the effect of human disturbance [13]. Finally, some pigeons may forage at night to avoid intraspecific competition [1,2].

Urban areas have a temporal stabilization of resources and environmental conditions, which induces a temporal persistence of species that thrive in cities [1]. This temporal persistence of species induces a temporal homogenization of bird composition, because the same species tend to be present during day and night, and different seasons and years [6,7,8,43,44]. This study showed that pigeon nocturnal foraging activity was related to artificial light. Therefore, efforts should be made to reduce light pollution in urban areas.

## 6. Conclusions

The present results showed that Feral Pigeon nocturnal activity is common in medium-sized and large cities of Argentina. Nocturnal activity was recorded in cities located at different altitudes and latitudes. However, more studies are needed to identify the environmental factors driving the biogeographical variation of nocturnal activity in Feral Pigeon, such as the city characteristics that promote nocturnal foraging. At the local scale, this study showed that artificial light was the main driver of nocturnal foraging. Other factors such as food availability should be addressed in future studies. 

## Figures and Tables

**Table 1:** Characteristics of cities surveyed and occurrence of nocturnal foraging by Feral Pigeons (*Columba livia f. domestica*) in the most highly urbanized areas of four medium-sized and big cities of Argentina.

City	Latitude	Altitude (m a.s.l.)	Population (2010 National Census)	Survey Length (km)	Nocturnal Foraging
Buenos Aires	34°35′ S	25	2,890,151	10.0	Yes
Rosario	32°57′ S	25	948,312	2.6	No
Mar del Plata	38°00′ S	27	765,000	3.3	Yes
Salta	24°47′ S	1187	535,303	1.8	Yes

**Table 2:** Description of environmental variables in sites with presence of foraging pigeons and random sites in Buenos Aires and Salta, Argentina. N: number of sites surveyed, SD: Standard deviation.

Variable	Buenos Aires				Salta			
	Pigeon (N = 8)		Random (N = 8)		Pigeon (N = 4)		Random (N = 4)	
	Mean	SD	Mean	SD	Mean	SD	Mean	SD
Mean lux	102.50	82.53	20.88	15.62	64.50	36.99	55.75	28.29
Pedestrians/3 min	43.63	16.86	25.25	16.82	193.75	144.35	154.75	106.01
Cars/3 min	61.63	36.93	22.38	12.18	0.00	0.00	70.50	108.98

**Table 3:** Generalized linear model explaining the occurrence of nocturnal foraging by Feral Pigeons (*Columba livia f. domestica*) in Buenos Aires and Salta, Argentina.

Variable	Estimate	Standard Error	z Value	*p*
Intercept	−1.835	0.925	−1.985	0.047
Lux	0.035	0.017	2.108	0.035
